# Nationwide real-world practice pattern and clinical data of palbociclib in HR (+), HER2 (−) metastatic breast cancer patients in Korea (KCSG BR21-15)

**DOI:** 10.1016/j.breast.2025.104500

**Published:** 2025-05-12

**Authors:** Jieun Lee, Dae-Won Lee, Min Hwan Kim, Jee Hung Kim, Ju Won Kim, Jae-Ho Byun, Kyoung Eun Lee, Myoung Joo Kang, Su-Jin Koh, Soojung Hong, Hye Sung Won, Han Jo Kim, In Hae Park, Seong Hoon Shin, Sun Kyung Baek, Seul-Gi Kim, Sung Ae Koh, Joo Young Jung, Ji-Yeon Kim, Gun Min Kim, Kabsoo Shin, In Sook Woo, Hyun Seon Kim, Seock-Ah Im, Yeon Hee Park

**Affiliations:** a Division of Medical Oncology, Department of Internal Medicine, Seoul St. Mary's Hospital, College of Medicine, The Catholic University of Korea, Seoul, Republic of Korea; b Department of Internal Medicine, Seoul National University Hospital, Seoul National University College of Medicine, Seoul, Republic of Korea; c Division of Medical Oncology, Department of Internal Medicine, Yonsei Cancer Center, Yonsei University College of Medicine, Seoul, Republic of Korea; d Division of Medical Oncology, Department of Internal Medicine, Gangnam Severance Hospital, Yonsei University, College of Medicine, Seoul, Republic of Korea; e Division of Hemato-Oncology, Department of Internal Medicine, Korea University College of Medicine, Anam Hospital, Republic of Korea; f Division of Medical Oncology, Department of Internal Medicine, Incheon St. Mary's Hospital, College of Medicine, The Catholic University of Korea, Seoul, Republic of Korea; g Department of Hematology and Oncology, Ewha Womans University Hospital, Seoul, Republic of Korea; h Division of Hematology and Oncology, Inje University Haeundae Paik Hospital, Busan, Republic of Korea; i Department of Hematology and Oncology, Ulsan University Hospital, Ulsan University College of Medicine, Republic of Korea; j Department of Internal Medicine, National Health Insurance Service Ilsan Hospi-tal, Goyang, Republic of Korea; k Division of Medical Oncology, Department of Internal Medicine, Uijeongbu St. Mary's Hospital, College of Medicine, The Catholic University of Korea, Seoul, Republic of Korea; l Division of Hematology and Oncology, Department of Internal Medicine, Soonchunhyang University Hospital, Republic of Korea; m Division of Hemato-Oncology, Department of Internal Medicine, Korea University College of Medicine, Guro Hospital, Republic of Korea; n Department of Internal Medicine, College of Medicine, Kosin University, Busan, Republic of Korea; o Department of Internal Medicine, Kyung Hee University Medical Center, Seoul, Republic of Korea; p Hematology and Oncology, Department of Internal Medicine, CHA Bundang Medical Center, CHA University, Seongnam, Republic of Korea; q Department of Hematology-Oncology, College of Medicine, Yeungnam University, Daegu, Republic of Korea; r Department of Hemato-oncology, Hallym University Dongtan Sacred Hospital, Hwaseong, Republic of Korea; s Division of Hematology-Oncology, Department of Medicine, Samsung Medical Center, Sungkyunkwan University School of Medicine, Seoul, Republic of Korea; t Division of Medical Oncology, Department of Internal Medicine, Yeouido St. Mary's Hospital, College of Medicine, The Catholic University of Korea, Seoul, Republic of Korea; u Pfizer Oncology, Seoul, Republic of Korea

**Keywords:** Hormone receptor–positive breast cancer, Palbociclib, Real-world data, Korea, Asia

## Abstract

**Background:**

Cyclin-dependent kinase (CDK) 4/6 inhibitors have remarkably improved the survival outcome in hormone-receptor-positive (HR+)/human epidermal growth factor-2-negative (HER2-) metastatic breast cancer (mBC). Although PALOMA-2 has met its primary outcome, overall survival (OS) was relatively shorter compared to ribociclib and abemaciclib. In Korea, use of palbociclib + aromatase inhibitor (AI) + gonadotropin-releasing hormone agonist (GnRHa) in premenopausal women is limited, and bilateral salpingo-oophorectomy (BSO) is necessary before treatment. We analyzed the real-world clinical outcome and patient characteristics of letrozole + palbociclib in Korea.

**Methods:**

Between August 2016 and December 2022, 1017 HR+/HER2-postmenopausal women treated with first-line letrozole + palbociclib were enrolled. Primary endpoints were real-world progression-free survival (rwPFS) in total population and survival differences according to menopausal status (natural or induced menopause via BSO).

**Results:**

Patients’ median age was 56 (range 27–92) years. Median rwPFS, real-world OS (rwOS) were 28.0 months (95 % confidence interval [CI] 25.5–32.1) and 61.8 months (95 % CI 57.7–70.5), with a median follow-up of 45.1 (IQR, 31.0–56.6) months. BSO group demonstrated similar median rwPFS compared to natural menopause group. Adjuvant tamoxifen ± GnRHa was most frequently prescribed (73.3 %). Primary endocrine resistant mBC patients showed inferior median rwPFS compared to secondary resistant mBC (14.6 vs. 27.1 months, *p* = 0.0063). Overall response rate was 47.5 %, with a disease control rate of 89.6 %.

**Conclusion:**

This is the largest country-based real-world study on palbociclib + letrozole in Asia. Palbociclib demonstrated median rwOS over 60 months, comparable to other pivotal trials.

## Highlights

• This is the real-world cohort data of first-line letrozole + palbociclib in HR+/HER2-mBC patients in Korea.
• Median rwPFS and rwOS were comparable to that of other pivotal phase III trials.
• Median rwPFS was comparable between premenopausal patients with BSO-induced menopause and those with natural menopause.
• Primary endocrine resistance showed inferior mPFS compared to secondary resistance.

## 1 Introduction

Breast cancer is the most prevalent cancer diagnosed among females worldwide [1], and is also the most common cancer among Korean women [2]. Hormone-receptor-positive (HR+)/human epidermal growth factor-2-negative (HER2-) metastatic breast cancer (mBC) accounts for approximately 70 % of all mBC in both Western and Asian countries [3,4]. However, the median age of Asian patients is approximately 45–49 years, which is lesser than the median age of 70 years in the Western population [5]. Considering the younger median age of diagnosis in Asia, approximately 40 % of Asian patients are diagnosed during the premenopausal stage, while approximately 20–30 % of these patients are premenopausal in Western countries [6,7]. Other than age and menopausal status, constant data suggest that biologic features are different between Western and Asian patients [8,9].

The combination of cyclin-dependent kinase 4/6 (CDK4/6) inhibitors with endocrine treatment dramatically improves survival outcomes in patients with HR+/HER2-mBC in terms of progression-free survival (PFS) and overall survival (OS). At present, the combination of CDK4/6 inhibitors with aromatase inhibitors (AI) is the standard first-line treatment for HR+/HER2-mBC patients [10,11] based on three pivotal clinical trials [[12], [13], [14]]. Palbociclib is the first-comer among the three CDK4/6 inhibitors, and PALOMA-2 showed prolonged PFS with letrozole + palbociclib compared to letrozole alone as the first-line treatment. Based on the PALOMA-2 trial, palbociclib was approved by the U.S Food and Drug Administration in February 2015 [15] and the Korean Ministry of Food and Drug Safety in August 2016 [16].

PALOMA-2 trial met its primary endpoint (PFS) but did not show a benefit in terms of OS [17]. Additionally, it showed a relatively shorter median OS compared to that of other pivotal phase III trials with ribociclib or abemaciclib [18,19]. However, PARSIFAL-LONG reported a median OS (mOS) of 61.9 months in letrozole + palbociclib-treated patients [20], and Young-PEARL demonstrated mOS of 54.8 months in premenopausal women, which is longer than that reported in the PALOMA-2 [21]. Therefore, a growing need exists to investigate the treatment pattern and survival outcome of palbociclib as first line treatment in various real-world settings.

Randomized controlled trials (RCT) have strengths based on the power of randomization and minimization of confounding bias. However, owing to the firm inclusion and exclusion criteria, the enrolled patients may differ somewhat from the general population. Although a risk of bias exists, population-based real-world studies reflect patients in routine practice and may focus on specific topics or long-term clinical outcomes, which are difficult to address in large-scale RCTs [22]. Large-scale real-world studies of palbociclib were conducted in Western, showing the superiority of palbociclib + AI compared to AI alone [[23], [24], [25]]. Palbociclib demonstrated its clinical benefit in Asian real-world studies as well, but the endocrine partners and treatment lines were heterogeneous [26,27].

In Korea, only ribociclib + AI + gonadotropin-releasing hormone agonist (GnRHa) is approved and reimbursed as first line treatment in premenopausal women, based on MONALEESA-7 [28,29]. Use palbociclib + AI + GnRHa as first line treatment in premenopausal women is limited, and bilateral salpingo-oophorectomy (BSO) is necessary before starting palbociclib based on the unique approval and reimbursement status. We previously reported that survival outcomes did not differ between patients experiencing natural menopause and those who underwent BSO in a pilot analysis [30]; however, a large-scale study is warranted to analyze the unique characteristics of these patients in Korea. Herein, we analyzed the real-world clinical outcomes of letrozole + palbociclib as first line treatment for HR+/HER2- Korean mBC patients, including distinct characteristics of Korean patients.

## 2 Patients and methods

### 2.1 Patients

Between August 2016 and December 2022, the medical records of HR+/HER2-postmenopausal women treated with letrozole + palbociclib as first line treatment were reviewed. A total of 1017 patients from 21 tertiary institutions participating in the Korean Cancer Study Group (KCSG) Breast Cancer Committee were enrolled. Patient data were collected mostly from clinical data warehouses (CDW) or retrospectively from a few institutions. The principal investigators or sub-investigators thoroughly reviewed the records for accurate data collection. Enrolled patients received at least one cycle of letrozole + palbociclib.

Menopause was defined in patients aged≥60 years or 12 consecutive months of amenorrhea in those aged <60 years. Patients who were premenopausal but received BSO for ovarian function suppression were also considered postmenopausal. Estrogen receptor (ER) and progesterone receptor (PR) positivity and HER2-negativity was defined based on the American Society of Clinical Oncology/College of American Pathologists guideline (ASCO/CAP guideline) [31,32]. Luminal A-like subtype was defined as ER and/or PR-positive, HER2 negative with a Ki-67 index ≤20 %. Luminal B-like subtype was defined as ER and/or PR-positive, HER2 negative with a Ki-67 index >20 % [33]. Primary endocrine resistance was defined as recurrence during the first 2 years of adjuvant tamoxifen or aromatase inhibitor treatment. Secondary endocrine resistance was defined as a recurrence during adjuvant tamoxifen or aromatase inhibitor but after the first 2 years, or within the first 12 months after completion of adjuvant endocrine treatment [34]. To reflect the unique characteristics of the patient population and adjuvant treatment patterns in Korea, we introduced the concept of “tamoxifen resistance” in the study. Among patients who received adjuvant tamoxifen, recurrence within the first 2 years was classified as primary tamoxifen resistance. In line with the definition of secondary endocrine resistance, recurrence occurring after the initial 2 years of adjuvant tamoxifen or within 12 months following its completion was classified as secondary tamoxifen resistance in our analysis.

### 2.2 Treatment schedule and response evaluation

Patients received 125 mg palbociclib orally for 3 weeks, followed by 1 week off schedule (4-week cycle). Letrozole was administered at 2.5 mg/day orally daily. Premenopausal patients underwent BSO before initiating palbociclib. Response evaluation was performed based on appropriate imaging studies, such as computed tomography scans or magnetic resonance imaging scans every three cycles, using the Response Evaluation Criteria in Solid Tumors (RECIST) criteria version 1.1. ^18^F-FDG positron emission tomography-computed tomography was also available for response evaluation based on physicians' clinical needs. Toxicity was assessed using the National Cancer Institute Common Terminology Criteria for Adverse Events, version 4.0, during each cycle. Treatment was administered until disease progression, unacceptable toxicity, or patients’ refusal.

### 2.3 Statistical analysis

Real-world PFS (rwPFS) was defined as the period from the first date of letrozole + palbociclib administration to the date of disease progression confirmed by imaging studies or patients' death. Real-world PFS2 (rwPFS2) was defined from the first date of letrozole + palbociclib administration to the progression date of subsequent line of therapy or death of any cause. Real-world OS (rwOS) was calculated from the start date of letrozole + palbociclib administration to patient death or to last follow-up date. Disease-free survival (DFS) was defined from the date of surgery to cancer recurrence, confirmed by imaging studies. Treatment-free interval (TFI) was defined from the completion date of any type of adjuvant endocrine treatment such as tamoxifen or AIs to the time of cancer recurrence. Overall response rate (ORR) was defined as the proportion of patients showing a complete response (CR) or partial response (PR) over the total patient population, based on RECIST v.1.1. Disease control rate (DCR) was defined as the ratio of patients showing CR, PR, or stable disease (SD) to the total patient population. Continuous variables are presented as median values, and categorical variables are presented as percentages. Continuous variables were compared using the Mann-Whitney *U* test, while categorical variables were compared using the chi-square or Fisher's exact tests. Survival analyses were performed using the Kaplan-Meier method and compared using the log-rank test. Hazard ratios (HRs) for rwPFS and rwOS were estimated using the Cox proportional hazards model with a 95 % confidence interval (CI). Two-sided p-values are presented for all analyses, with p < 0.05 considered statistically significant. R ver. 4.4.1 (R Foundation for Statistical Computing, Vienna, Austria) was used for the statistical analyses.

## 3 Results

### 3.1 Patient characteristics

**Supplementary Fig. 1:**
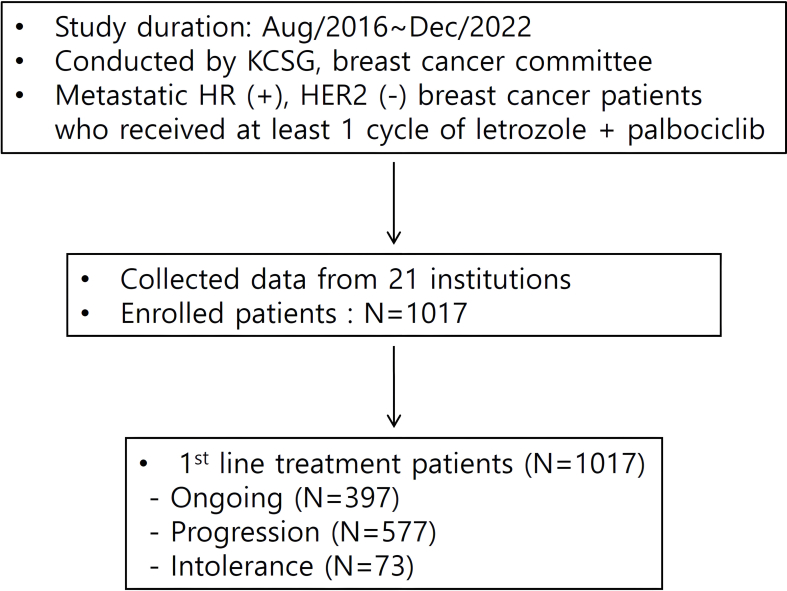
Consort diagram.

Between August 2016 and December 2022, 1017 HR+/HER2-patients who received first line letrozole + palbociclib were enrolled (Supplementary Fig. 1). The median follow-up duration was 45.1 months (interquartile range 31.0–56.6 months). Table 1 summarizes the baseline patient characteristics. The median age was 56 years (range, 27–92 years), which was slightly younger than that of the PALOMA-2 population. Approximately 40 % of the patients were classified as luminal A-like subtype. Over one-third (383 patients, 37.7 %) of the patients were initially diagnosed with *de novo* stage IV disease. Among stage I–III breast cancer, approximately 80 % of patients received neoadjuvant or adjuvant chemotherapy. All patients were postmenopausal, and 349 (34.3 %) patients underwent BSO for induced menopause prior to initiation of palbociclib. Among the total patient population, 191 (18.9 %) patients were diagnosed with bone-only disease, more than half of the patients (58.1 %) with visceral disease, and 190 (18.8 %) patients presented with liver metastasis. The exact incidence of germline Breast Cancer Susceptibility Gene 1/2 (*gBRCA1/2*) mutations is largely unknown because most patients (73.5 %) did not undergo *gBRCA1/2* mutation study. Among 270 (26.5 %) patients who underwent the *gBRCA1/2* mutation study, 28 (10.4 %) harbored *gBRCA1/2* mutation.

**Table 1:** Baseline patient characteristics.

	Patients (%)
**Patients**	1017
**Age (years)**	
median, range	56 (27–92)
<40	51 (5)
40∼64	725 (71.3)
65∼75	164 (16.1)
>75	77 (7.6)
**ECOG**	
0∼1	585 (57.5)
2∼3	309 (30.3)
Not assessed	123 (12.1)
**Histology**	
Invasive ductal carcinoma	756 (74.4)
Invasive lobular carcinoma	125 (12.3)
Invasive papillary carcinoma	23 (2.3)
Mucinous carcinoma	19 (1.9)
others	13 (1.3)
Not assessed	80 (7.9)
**Estrogen Receptor**	
Positive	939 (92.3)
Negative	22 (2.2)
Not assessed	56 (5.5)
**Progesterone receptor**	
Positive	769 (75.6)
Negative	190 (18.7)
Not assessed	58 (5.7)
**Luminal**	
A-like	434 (42.7)
B-like	340 (33.4)
Unknown	243 (23.9)
**Stage at initial diagnosis**	
I	108 (10.6)
II	254 (25.0)
III	211 (20.7)
IV	383 (37.7)
Not assessed	61 (6.0)
**Prior neoadjuvant and adjuvant chemotherapy**	517 (50.8)
Neoadjuvant and adjuvant chemotherapy	37 (3.6)
Neoadjuvant chemotherapy	83 (8.2)
Adjuvant chemotherapy	397 (39.0)
Not done	105 (10.3)
Not assessed	12 (1.2)
**Method of menopause**	
Natural menopause	657 (64.6)
Menopause after BSO	349 (34.3)
Not assessed	11 (1.1)
**Presence of visceral metastasis**	
Nonvisceral	422 (41.5)
Asymptomatic visceral	478 (47.0)
Symptomatic visceral	113 (11.1)
Not assessed	4 (0.4)
**Bone only metastasis**	
Yes	191 (18.9)
No	822 (81.1)
**Liver metastasis**	
Yes	190 (18.8)
No	823 (81.2)
**Lung metastasis**	
Yes	396 (39.1)
No	617 (60.9)
**Brain metastasis**	
Yes	46 (4.5)
No	967 (95.5)
**Palbociclib combined after prior letrozole**	
Yes	28 (2.8)
No	989 (97.2)
**Familial history of cancer**	
No	663 (65.2)
Yes	210 (20.6)
Breast	82 (8.1)
Ovary	4 (0.4)
Pancreas	7 (0.7)
Prostate	5 (0.5)
Others	116 (54.2)
Not assessed	144 (11.4)
**gBRCA status**	
Not detected	211 (20.8)
*BRCA1*	5 (0.5)
*BRCA2*	23 (2.3)
*BRCA1* or *BRCA2* VUS	19 (2.9)
*PALB2*	1 (0.1)
Not assessed	747 (73.5)

Patients with recurrent breast cancer were relatively younger than *de novo* mBC patients, and there was no statistical difference in the rate of natural or induced menopause between the two groups (Supplementary Table S1). Among patients with recurrent breast cancer, most received adjuvant endocrine treatment (550 patients, 87.9 %), with a completion rate of 41.5 %. The median duration of adjuvant endocrine treatment was 4.4 years. Overall, 35 % of the patients did not complete adjuvant endocrine treatment due to early cancer recurrence, and 12.3 % dropped adjuvant endocrine treatment due to intolerance. Tamoxifen was the most commonly prescribed adjuvant endocrine therapy (*n* = 374, 68 %). Among 285 patients (45 %) who showed primary or secondary endocrine resistance to adjuvant tamoxifen or aromatase inhibitor based on ESMO guideline [34], most patients (267 patients, 41.5 %) were treated with tamoxifen. About 30 % of endocrine resistance patients showed primary tamoxifen resistance, 61.4 % of patients with secondary tamoxifen resistance (Table 2) [34].

**Table 2:** Patient characteristics among recurrent disease.

	Patients (%)
**Patients**	633
**Menopausal state at initial cancer diagnosis**	
Premenopausal	386 (61.0)
Postmenopausal	232 (36.7)
Not assessed	15 (2.4)
**Menopausal state at palbociclib administration**	
Postmenopausal	
Natural menopause	399 (63.0)
Prior BSO	225 (35.5)
Not assessed	9 (1.5)
**Adjuvant endocrine treatment**	
Refused	49 (7.8)
Yes	550 (87.9)
Not assessed	27 (4.3)
**Adjuvant endocrine regimen**	
Tamoxifen or toremifene	374 (68.0)
Tamoxifen + GnRHa	29 (5.3)
Aromatase inhibitor	102 (18.5)
Tamoxifen followed by aromatase inhibitor	28 (5.1)
Aromatase inhibitor followed by tamoxifen	4 (0.7)
GnRHa monotherapy	1 (0.2)
Not assessed	12 (2.2)
**Duration of endocrine treatment (year)**	
Median duration (range)	4.4 (0.09–12.77)
**Completion of adjuvant endocrine treatment**	
No	304 (55.3)
intolerance	78 (12.3)
recur	226 (35.7)
Yes	228 (41.5)
Not assessed	18 (3.3)
**Endocrine resistance**	
No	332 (52.4)
Yes	285 (45.0)
Primary tamoxifen resistance	92 (32.3)
Secondary tamoxifen resistance	175 (61.4)
Secondary resistance to aromatase inhibitor	18 (6.3)
Not assessed	16 (2.5)
**Disease-free survival**	
<12 months	27 (4.3)
≥12 months	597 (95.7)
**Treatment-free interval**	
<12 months	274 (51.5)
≥12 months	258 (48.5)

Most patients (87 %, 543) had a median DFS (mDFS) of >24 months. Regarding the TFI, 41.4 % of patients presented with a TFI >24 months, and 51.5 % of patients presented with a TFI less than 12 months (Table 2).

### 3.2 Efficacy

**Fig. 1:**
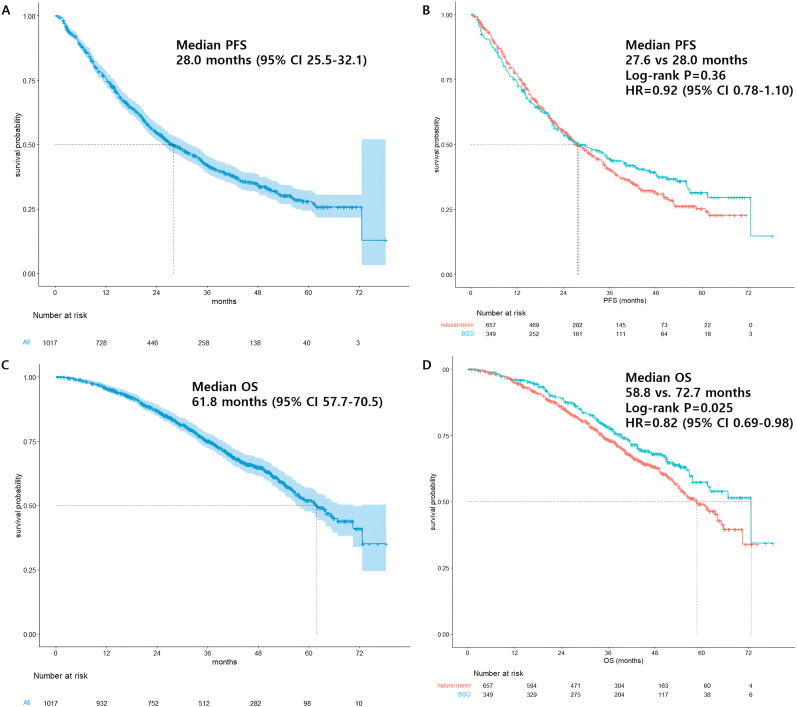
Survival Outcomes. (A) Median PFS in total patient population. (B) Median PFS according to method of menopause. (C) Median OS of total patient population. (D) Median OS according to method of menopause.

**Supplementary Fig. 2:**
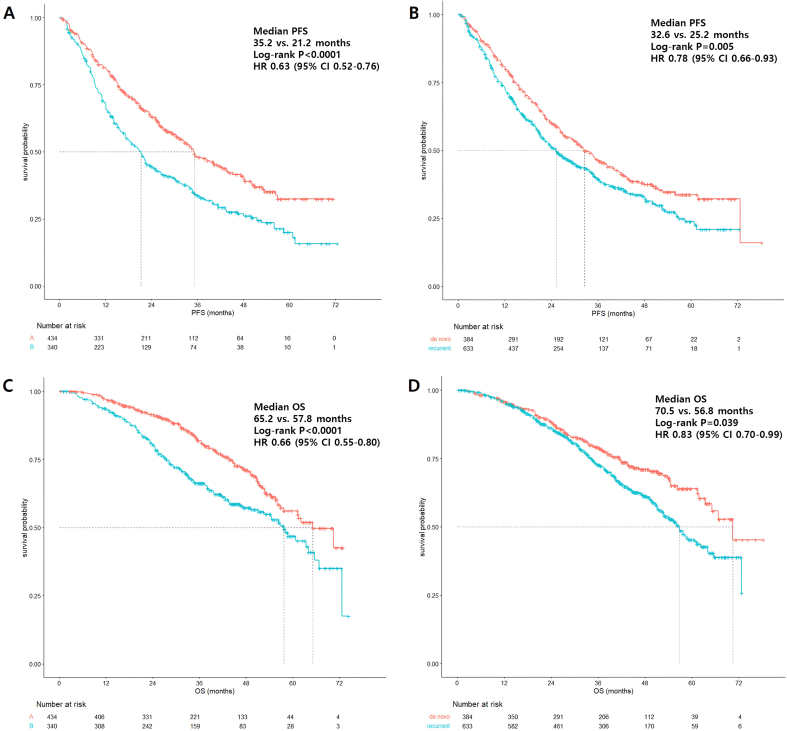
Survival outcomes according to luminal subtype or recurrence status. (A) Median PFS according to luminal A or B-like subtype. (B) Median PFS according to *De novo* or recurrent breast cancer status. (C) Median OS according to luminal subtype. (D) Median OS according to recurrent status.

**Supplementary Fig. 3:**
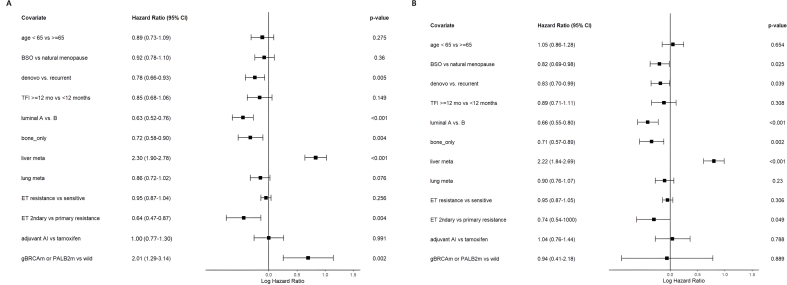
Forest plot in total patient population. (A) Subgroup analysis of PFS. (B) Subgroup analysis of OS.

The median rwPFS of letrozole + palbociclib was 28.0 months (95 % CI 25.5–32.1 months) (Fig. 1A). There were no statistical difference of median rwPFS according to natural or induced menopause (Fig. 1B). According to subtype, luminal A-like patients showed superior median rwPFS compared to luminal B-like subtype patients (35.2 months vs. 21.3 months, HR = 0.63, *p* < 0.0001). Patients who were diagnosed with *de novo* mBC demonstrated better median rwPFS compared to recurrent breast cancer patients (3 2.6 months vs. 25.2 months, HR = 0.78, *p* = 0.005) (Supplementary Fig. 2A and B). Patients with bone-only metastases showed favorable survival, while those with liver metastases showed poor outcome. Patients with *gBRCA1/2* or *PALB2* mutations demonstrated inferior PFS compared to patients without germline mutations (Supplementary Fig. 3A).

The median rwOS of enrolled patients was 61.8 months (95 % CI 57.73–70.53 months) (Fig. 1C). There was no statistical difference of median rwPFS between natural or induced menopause. However, patients who underwent BSO before starting palbociclib showed superior median rwOS compared to natural menopause breast cancer patients (72.7 months vs. 58.8 months, *p* = 0.025) (Fig. 1D). Luminal A-like breast cancer patients showed longer median rwOS compared to luminal B-like breast cancer (65.2 vs. 57.8 months, *p* < 0.0001), and *de novo* mBC patients showed superior median rwOS compared to the recurrent breast cancer patient subgroup (70.5 months vs. 56.8 months, *p* = 0.0022) (Supplementary Fig. 2C and 2D). Similar to the rwPFS data, patients with bone-only metastasis showed superior rwOS, and patients with liver metastasis showed poor rwOS outcomes. Patients with *gBRCA1/2* or *PALB2* mutations showed similar rwOS to patients without germline mutations (Supplementary Fig. 3B).

The ORR and DCR were 47.5 % and 89.6 %, respectively (Supplementary Table S2).

### 3.3 Survival according to endocrine resistance and TFI in recurrent patients

**Fig. 2:**
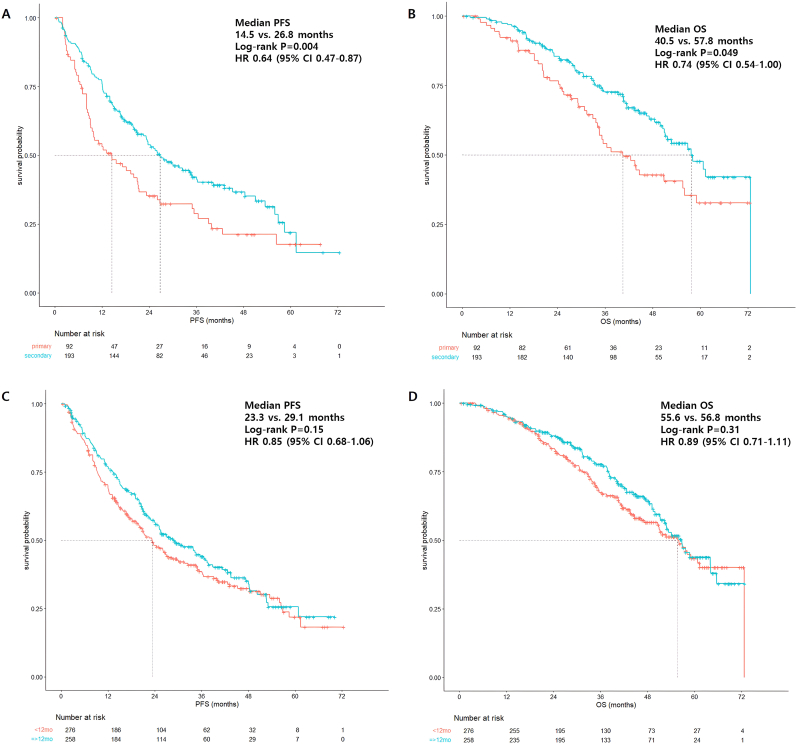
Survival outcomes according to endocrine resistance and TFI in recurrent patient population. (A) Median PFS according to primary or secondary endocrine resistance. (B) Median OS according to endocrine resistance. (C) Median PFS according to TFI <12 months or ≥ 12 months. (D) Median OS according to TFI.

Among patients with recurrent breast cancer, patients with secondary endocrine resistance demonstrated superior median rwPFS and median rwOS, compared to those with primary endocrine resistance (median rwPFS 26.8 vs. 14.5 months, *p* = 0.004; median rwOS 57.8 vs. 40.5 months, *p* = 0.049) (Fig. 2A and B). Trends for improvement of median rwPFS and median rwOS were noted in patients with TFI ≥12 months, but without statistical significance (Fig. 2C and D).

**Fig. 3:**
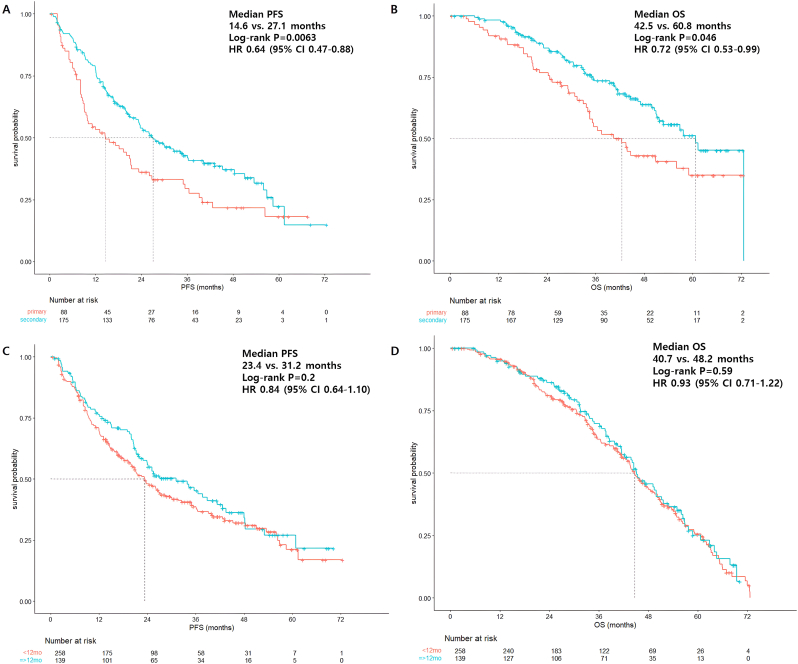
Survival outcomes according to tamoxifen resistance and TFI in recurrent patient population who received adjuvant tamoxifen. (A) Median PFS according to primary or secondary tamoxifen resistance. (B) Median OS according to tamoxifen resistance. (C) Median PFS according to TFI < 12 months or ≥ 12 months. (D) Median OS according to TFI.

We performed a subgroup analysis based on previous adjuvant tamoxifen or AI treatment in patients with recurrence. Considering that a large proportion of patients were pre-treated with adjuvant tamoxifen, TFI was defined from the completion of treatment with adjuvant tamoxifen or AI to the date of disease recurrence. In the adjuvant tamoxifen pretreatment group, patients with secondary tamoxifen resistance demonstrated longer median rwPFS and median rwOS than those with primary resistance (Fig. 3A and B). Regarding TFI in the tamoxifen-pretreated group, those with a TFI of ≥12-month showed trends for better median rwPFS and median rwOS, but without statistical significance compared to those with a TFI of <12-month (Fig. 3C and D).

### 3.4 Post-progression treatment and PFS2

In our study, post-treatment and rwPFS2 data were analyzed in patients who demonstrated disease progression during treatment with letrozole + palbociclib. Among 577 patients who showed disease progression, post-treatment data were collected from 547 patients. In this subgroup, the median rwPFS was 15.1 months (95 % CI 14.1–16.7 months). More than half of the patients (315, 57.6 %) received endocrine treatment, and the most common endocrine treatment was everolimus with exemestane (142, 25.9 %). Approximately one-third of the patients (206, 37.7 %) received cytotoxic chemotherapy, and capecitabine was the most commonly prescribed (195 patients, 35.6 %) (Table 3).

**Table 3:** Post-treatment after progression of letrozole plus palbociclib.

	Patients (%)
**Total patients**	547
**Cytotoxic chemotherapy**	206 (37.7)
**Conventional Chemotherapy**	195 (35.6)
Capecitabine	96 (17.6)
weekly paclitaxel	50 (9.1)
docetaxel	13 (2.4)
Anthracycline-based	12 (2.2)
Others	24 (4.4)
**Clinical trial_chemo**	7 (1.3)
**Clinical trai_PARP inhibitor**	3 (0.5)
**Talazoparib**	1 (0.2)
**Endocrine treatment**	315 (57.6)
**conventional Endocrine treatment**	263 (48.1)
fulvestrant	112 (20.5)
Everolimus + Exemestane	142 (25.9)
alpelisib + fulvestrant	1 (0.2)
others	8 (1.5)
**Abemaciclib**	2 (0.4)
**Clinical trial_endo**	50 (9.1)
**BSC or FU loss**	26 (4.8)

**Supplementary Fig. 4:**
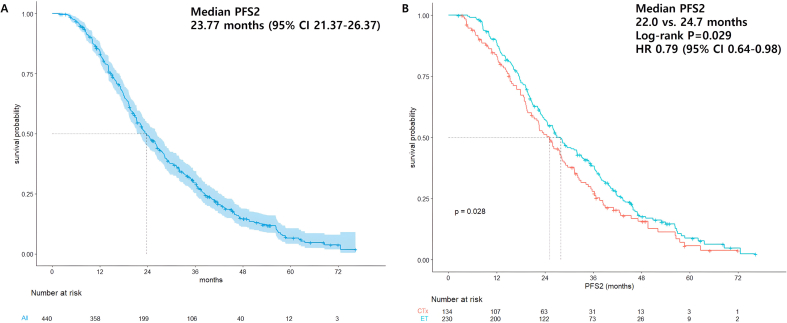
(A) Median PFS2 among patients who received second-line treatment after progression of letrozole + palbociclib. (B) Median PFS2 according to second-line cytotoxic chemotherapy or endocrine treatment.

The median rwPFS2 was 23.8 months (95 % CI 21.4–26.4 months). Chemotherapy-treated patients showed inferior median rwPFS2 compared to endocrine-treated patients (median rwPFS2, 22.0 months vs. 24.7 months, *p* = 0.028) (Supplementary Fig. 4). This result might be due to the selection of chemotherapy in the patient subgroup with aggressive clinicopathological characteristics compared with the endocrine-treated patient subgroup.

### 3.5 Dose adjustment and safety

Most patients received an initial dose of 125 mg as the starting dose. Approximately half of the patients underwent one dose reduction to 100 mg, with a median 3 cycles of palbociclib before dose reduction (Supplementary Table S3). The most common adverse event resulting in dose reduction was neutropenia. Patients tolerated the treatment with more than 90 % drug continuation without dropping out during treatment.

The most common adverse event was neutropenia of any grade, and 82.5 % of the patients experienced grade 3 or 4 neutropenia. Approximately one-third of the patients complained of fatigue of any grade (Supplementary Table S4). No new safety signals have been reported compared to previous clinical trials.

## 4 Discussion

In this study, we aimed to analyze the real-world survival outcomes and unique characteristics of patients treated with letrozole + palbociclib in routine clinical practice in Korea. Our study revealed outstanding survival outcomes, with median rwPFS of 28.0 months and median rwOS of 61.83 months, which are comparable to the survival outcomes with the use of other CDK4/6 inhibitors. Furthermore, the survival data are consistent with previously reported real-world data (RWD) in Western countries and Japan [23,27,35]. To the best of our knowledge, this is the largest RWD of homologous first-line letrozole + palbociclib in the Asian region.

Before the approval of ribociclib and abemaciclib, palbociclib was the only CDK4/6 inhibitor used as first- or second-line treatment in Korea. At that time, there were substantial limitations in the selection of endocrine treatment for HR+/HER2-premenopausal mBC women. PALOMA-2 demonstrated profound median PFS with first-line treatment, but this trial had a critical weakness in omitting premenopausal women from the inclusion criteria. Palbociclib granted FDA approval only in postmenopausal women, and many other countries including Korea approved palbociclib only in postmenopausal women based on the suboptimal inclusion criteria of PALOMA-2. Therefore, premenopausal women had limitations in using CDK4/6 inhibitors in the first-line setting, although many clinicians assumed that palbociclib may have a similar magnitude of benefit. Administration of AI + GnRHa in premenopausal patients has been approved in Korea since 2017, and they only had a choice of cytotoxic chemotherapy, except tamoxifen, as a systemic treatment before this approval. Based on approval status, adjuvant or palliative tamoxifen was mandatory before starting AI + GnRHa in premenopausal women. A lack of evidence and the absence of approval from authorities had driven many premenopausal patients to receive cytotoxic chemotherapy irrespective of the disease burden. To use palbociclib as a first-line treatment in premenopausal women, they had to be postmenopausal status before treatment. Therefore, many premenopausal women had to undergo BSO to become postmenopausal before starting palbociclib. This is one of the biggest differences in Korea compared to other RWDs.

This unmet medical need of premenopausal women came into focus based on the positive results of the MONALEESA-7, which showed significant survival benefit of AI + ribociclib in premopausal women [28]. Young-PEARL also focused on premenopausal patients and proved the survival benefit of palbociclib + AI compared to capecitabine. Based on Young-PEARL, FDA expanded the approval of palbociclib to premenopausal women in December 2022 [36]. However, many premenopausal women still have limitations in assessing palbociclib in real-world practice due to approval issue in each country. Based on previous trials, the Advanced Breast Cancer International Consensus recommended that future trials should include both premenopausal and postmenopausal women and that premenopausal women should be treated equally to postmenopausal women [37].

In our study, 34.3 % of patients were premenopausal and underwent BSO before starting letrozole + palbociclib. Therefore, a relatively higher proportion of included patients were younger than those in the PALOMA-2. Moreover, our study showed different recurrence pattern in those with luminal-like breast cancer; 35 % recurred on adjuvant endocrine treatment with shorter DFS and comprised a higher endocrine-resistant population, which is the most important discriminating finding to consider compared with other Western populations. Compared to PALOMA-2, more patients had a TFI of <12 months (51.5 % vs. 22.3 %), representing a more resistant tumor biology to endocrine treatment. PALOMA-2 enrolled endocrine-sensitive patients with TFI >12 months after the completion of adjuvant AI. Exceptionally, approximately 10 % of tamoxifen-pretreated patients who showed progression during adjuvant treatment or within 1 year of TFI were enrolled in the PALOMA-2 [12,38]. In Korea, the approval of palbociclib was based on the inclusion criteria of the PALOMA trials. Therefore, tamoxifen-pretreated postmenopausal women who showed disease progression during adjuvant treatment or TFI <1 year could receive AI + palbociclib. Despite these different patient profiles, our data showed consistent median rwPFS compared with the long-term follow-up data of the PALOMA-2 trial and a median rwOS of over 60 months.

In this study, no difference in median rwPFS was observed based on natural menopause or induced menopause (BSO). However, superior median rwOS was observed in the BSO group. This is in line with Spain's RWD, which showed a similar rwPFS irrespective of menopausal status and a superior rwOS in premenopausal group [39]. These data might suggest that palbociclib may play a role irrespective of patients' age and molecular tumor biology. MONALEESA-7 which enrolled premenopausal women exclusively, ribociclib + AI also showed a durable extension of mPFS and OS [28,29]. Even though this may be a hypothetical theory, CDK4/6 inhibitors, including palbociclib, may play a role irrespective of menopausal status, patients' age, and molecular biology.

Recently, superior rwPFS2 was reported in P-REALITY X trial, but a detailed post-treatment regimen was not reported [40]. In our study, post-treatment regimens and rwPFS2 were reviewed in detail. Although guidelines suggest subsequent endocrine treatment unless the patient does not show endocrine resistance [10,11], approximately 40 % of the patients in this study received cytotoxic chemotherapy as a subsequent line of treatment. This preference for chemotherapy might be due to the relatively short mPFS of standard endocrine treatment after the progression of CDK4/6 inhibitors [41]. Among patients who received cytotoxic chemotherapy, capecitabine was the most preferred regimen. This preference is based on the data of Young-PEARL, in which capecitabine showed an mPFS of up to 14 months [21]. As fulvestrant monotherapy shows an mPFS of approximately 3 months and alpelisib is not reimbursed with a high risk of adverse events [41,42], everolimus plus exemestane was the most preferred regimen in patients receiving endocrine treatment. The median rwPFS2 in this study was 23.8 months, which is relatively shorter than that in the P-REALITY X trial, implying more aggressive tumor behavior in this dataset. Although median rwPFS2 was relatively short, median rwOS was durable in the analysis, indicating the importance of early palbociclib administration. More long-term follow-ups are warranted to estimate mPFS2 levels in the clinical setting.

This study included postmenopausal women treated with first-line regimen of letrozole + palbociclib homogeneously. Palbociclib demonstrated a sustained survival benefit irrespective of the type of endocrine resistance. In addition to favorable median rwPFS, our study reported a durable median rwOS of >60 months, which is longer than that reported in pivotal PALOMA-2 and comparable to PARSIFAL-LONG. Although MONALEESA7 used different CDK4/6 inhibitor (ribociclib) for the study, it was an only trial dedicated for premenopausal women and the endocrine resistance pattern may be similar to our patient cohort. Within line of superior survival outcome of AI + CDK4/6 inhibitor over AI alone in MONALEESA7, the favorable survival outcome of AI + palbociclib was reproduced in our real-world study.

We estimated the following potential factors that may have contributed to superior survival outcomes in our analysis. A higher proportion of patients with *de novo* mBC may have also contributed to this prolonged survival; in our cohort, *de novo* mBC accounted for approximately 38 %, compared to 32 % in PALOMA-2. In our subgroup analysis, *de novo* patients showed statistically superior survival outcomes and these may influenced the durable rwPFS and rwOS. In addition, the younger age distribution in our cohort led to a higher proportion of patients receiving adjuvant tamoxifen without previous aromatase inhibitor exposure when compared to PALOMA-2 trial. Most recurrent breast cancer patients in our cohort had been pretreated with tamoxifen and exhibiting primary or secondary tamoxifen resistance without history of AI exposure. This unique treatment background, which differs from other patient populations, might have influenced the favorable survival outcome observed in our patient cohort. Lastly, the distinct biological characteristics of Asian breast cancer patients [5,9], compared to their Western counterparts, may also have contributed to the observed outcomes.

The primary limitation of this study is its retrospective nature. Although most data were collected from the CDW, some data were collected in retrospective manner in few institutions. To overcome the limitation of retrospective study, authors have intensively reviewed the electronic medical records of each enrolled patient and rechecked the missing data to enhance the accuracy of the collected medical data. Another limitation is the lack of a control arm, which may be a reference to interpretate the survival outcome of the study. Finally, comparing the survival outcome with PALOMA-2 should be interpretated with caution considering PALOMA-2 trial is based on randomized, control study which controlled heterogeneous bias during analysis. Nevertheless, our study has the strength of being the largest real-world Asian dataset of homogeneous first-line treatment with letrozole + palbociclib in postmenopausal patients with HR+/HER2-metastatic breast cancer.

## 5 Conclusion

This study analyzed the unique patient characteristics and survival outcome of first-line letrozole + palbociclib as nationwide, real-world data analysis. This data holds its significance as a largest real-world dataset reported from Asia. We demonstrated a durable rwOS outcome of over 60 months, comparable to that of other pivotal phase III clinical trials. Furthermore, many patients who had undergone BSO, which is a unique Korean patient population, were included in this study, and this patient characteristic cannot be reproduced in other countries. These findings can be one of a valuable reference to letrozole + palbociclib as first-line treatment in real-world patient population.

## CRediT authorship contribution statement

**Jieun Lee:** Writing – review & editing, Writing – original draft, Validation, Methodology, Investigation, Funding acquisition, Formal analysis, Data curation, Conceptualization. **Dae-Won Lee:** Writing – review & editing, Data curation. **Min Hwan Kim:** Writing – review & editing, Data curation. **Jee Hung Kim:** Writing – review & editing, Data curation. **Ju Won Kim:** Writing – review & editing, Data curation. **Jae-Ho Byun:** Writing – review & editing, Data curation. **Kyoung Eun Lee:** Writing – review & editing, Data curation. **Myoung Joo Kang:** Writing – review & editing, Data curation. **Su-Jin Koh:** Writing – review & editing, Data curation. **Soojung Hong:** Writing – review & editing, Data curation. **Hye Sung Won:** Writing – review & editing, Data curation. **Han Jo Kim:** Writing – review & editing, Data curation. **In Hae Park:** Writing – original draft, Data curation. **Seong Hoon Shin:** Writing – review & editing, Data curation. **Sun Kyung Baek:** Writing – review & editing, Data curation. **Seul-Gi Kim:** Writing – review & editing, Data curation. **Sung Ae Koh:** Writing – review & editing, Data curation. **Joo Young Jung:** Writing – review & editing, Data curation. **Ji-Yeon Kim:** Writing – review & editing, Data curation. **Gun Min Kim:** Writing – review & editing, Data curation. **Kabsoo Shin:** Writing – review & editing, Data curation. **In Sook Woo:** Writing – review & editing, Data curation. **Hyun Seon Kim:** Writing – review & editing. **Seock-Ah Im:** Writing – review & editing, Supervision. **Yeon Hee Park:** Writing – review & editing, Writing – original draft, Validation, Supervision, Methodology, Investigation, Data curation, Conceptualization.

## Ethical approval and informed consent

The study was performed in accordance with the Declaration of Helsinki. This study was approved by the institutional review board of each participating institution. The requirement for informed consent was waived because of the retrospective nature of this study.

## Consent to publication

All authors have read the paper and consented the publication.

## Access to data and data analysis

Jieun Lee has full access to all data in the study and takes responsibility for the analysis of the data. Data will be available from Jieun Lee (befamiliar@catholic.ac.kr) on reasonable request after approval or a proposal.

## Disclosure

The Co-author HS Kim was a former employee of Pfizer Korea, Seoul, Republic of Korea. All other authors have declared no conflicts of interest.

## Funding

This work was supported by Pfizer Korea, Seoul, Republic of Korea. The research was supported (in part) by the Korean Cancer Study Group. Additionally, this study was supported by the National R&D Program for Cancer Control through the 10.13039/501100003645National Cancer Center (10.13039/501100003645NCC) funded by the 10.13039/501100003625Ministry of Health & Welfare, Republic of Korea (HA22C0012).

## Glossary

AI: aromatase inhibitors
BSO: bilateral salpingo-oophorectomy
CDK4/6: cyclin-dependent kinase 4/6
CDW: clinical data warehouses
CR: complete response
DCR: disease control rate
DFS: disease-free survival
ER: estrogen receptor
GnRHa: gonadotropin-releasing hormone agonist
HR+/HER2: Hormone receptor-positive/human epidermal growth factor receptor 2-negative;
KCSG: Korean Cancer Study Group ORR, overall response rate
OS: overall survival
PFS: progression-free survival
PR: progesterone receptor
PR: partial response
RCT: Randomized controlled trials
RECIST: Response Evaluation Criteria in Solid Tumors
SD: stable disease
TFI: treatment-free interval
