# Supplementary material for: Nationwide real-world practice pattern and clinical data of palbociclib in HR (+), HER2 (−) metastatic breast cancer patients in Korea (KCSG BR21-15)
Source: Breast. 2025 May 12;82:104500. doi: 10.1016/j.breast.2025.104500 (PMC12100939; doi:10.1016/j.breast.2025.104500)
Supplement: Multimedia component 1 [file mmc1.docx]

**Supplementary Table 1. Age and menopause state in De novo and recurrent patients’ subgroup**

|  | De novo | Recurrent | P-value |
| --- | --- | --- | --- |
| No. of patients | N=384 | N=633 |  |
| Age (yr) |  |  |  |
| Median (range) | 57.0 [27~92] | 55.0 [29~90] | *0.006* |
| <40 | 22 ( 5.7%) | 29 ( 4.6%) |  |
| 40~64 | 260 (67.7%) | 465 (73.5%) |  |
| 65~75 | 62 (16.1%) | 102 (16.1%) |  |
| > 75 | 40 (10.4%) | 37 ( 5.8%) |  |
| **Menopausal state at palbociclib administration** | |  | 0.369 |
| Postmenopausal |  |  |  |
| Natural menopause | 258 (67.2%) | 399 (63.0%) |  |
| Prior BSO | 124 (32.3%) | 225 (35.5%) |  |
| Not assessed | 2 ( 0.6%) | 9 (1.5%) |  |

**Supplementary Table 2. Clinical response of letrozole plus palbociclib**

|  | **Total** |
| --- | --- |
| **Best response (n,%)** |  |
| Partial response | 483 (47.5%) |
| Stable disease | 428 (42.1%) |
| Progressive disease | 91 ( 8.9%) |
| Not assessed | 15 ( 1.5%) |
|  |  |
| **Overall response rate (n,%)** | 483 (47.5%) |
| **Disease control rate (n,%)** | 911 (89.6%) |

**Supplementary Table 3. Dose reduction of palbociclib**

|  | Patient number (%) |
| --- | --- |
| **No. of administered cycle (median, range)** | 21.0 [1~87] |
| Starting dose |  |
| 125mg | 908 (89.3) |
| 100mg | 101 ( 9.9) |
| 75mg | 7 ( 0.7) |
| Not assessed | 1 ( 0.1) |
| **first dose reduction** |  |
| No | 491 (48.3) |
| Yes | 526 (51.7) |
| median 1st dose reduction cycle | 3.0 [2~69] |
| **Second dose reduction** | 75 (7.4) |
| median 2nd dose reduction cycle | 8.0 [2~59] |
| **Discontinuation due to intolerance** |  |
| No | 944 (92.8) |
| Yes | 73 ( 7.2) |

**Supplementary Table 4. Treatment related adverse events**

|  | **Any grade** | **Grade 3** | **Grade 4** |
| --- | --- | --- | --- |
| **Hematologic** |  |  |  |
| Anemia | 640 (62.9) | 64 ( 6.3) | 0 |
| Neutropenia | 967 (95.1) | 638 (62.7) | 201 (19.8) |
| Thrombocytopenia | 483 (47.5) | 48 ( 4.7) | 11 ( 1.1) |
| **Non-hematologic** |  |  |  |
| Nausea | 133 (13.1) | 11 ( 1.1) | 0 |
| Mucositis | 282 (27.7) | 8 ( 0.8) | 0 |
| Diarrhea | 62 (6.1) | 0 | 0 |
| Fatigue | 340 (33.5) | 13 ( 1.3) | 0 |
