# Supplementary material for: Nationwide real-world practice pattern and clinical data of palbociclib in HR (+), HER2 (−) metastatic breast cancer patients in Korea (KCSG BR21-15)
Source: Breast. 2025 May 12;82:104500. doi: 10.1016/j.breast.2025.104500 (PMC12100939; doi:10.1016/j.breast.2025.104500)
Supplement: Multimedia component 2 [file mmc2.docx]

**Supplementary Fig. 1.** Consort diagram

**Supplementary Fig. 2.** Survival outcomes according to luminal subtype or recurrence status. (A) Median PFS according to luminal A or B-like subtype. (B) Median PFS according to *De novo* or recurrent breast cancer status. (C) Median OS according to luminal subtype. (D) Median OS according to recurrent status.

**Supplementary Fig. 3.** Forest plot in total patient population. (A) Subgroup analysis of PFS. (B) Subgroup analysis of OS.

**Supplementary Fig. 4.** (A) Median PFS2 among patients who received second-line treatment after progression of letrozole + palbociclib. (B) Median PFS2 according to second-line cytotoxic chemotherapy or endocrine treatment.
